# Cholangioscope-assisted endoscopic retrograde appendicitis therapy for the diagnosis and treatment of occult appendicitis with a giant fecalith

**DOI:** 10.1055/a-2514-2643

**Published:** 2025-02-05

**Authors:** Xia Peng, Yang Yu, Rengyun Xiang, Faliang Xiang, Zhi Peng, Xuefeng Li

**Affiliations:** 1 74680 Department of Gastroenterology, First Affiliated Hospital of Jishou University, Jishou, China

As a main cause of appendicitis, fecaliths have been identified in approximately 40% of patients with acute appendicitis 11 . Appendicoliths are usually less than 1 cm in their largest dimension, and those that are larger than 2 cm are termed giant appendicoliths 22 . The most common imaging modalities used to diagnose appendicitis are ultrasound and computed tomography (CT). Because its lumen is long and thin, the appendix can vary greatly, and therefore the basic reliance on ultrasound and CT is not always sufficient to make a reliable diagnosis of appendicitis 33 . Currently, the diagnosis of appendicitis remains challenging. Herein, we report a rare case of a pediatric patient with occult appendicitis. By means of cholangioscope-assisted endoscopic retrograde appendicitis therapy (ERAT), a giant appendicolith was discovered and flushed out of the appendiceal cavity.

A 13-year-old boy was admitted to our hospital with a 2-day history of right lower abdominal pain. Both ultrasound ( Fig. 1Fig. 1 **a** ) and low-dose CT ( Fig. 1Fig. 1 **b** ) revealed his appendix was normal. As he had persistent lower right abdominal pain, colonoscopy and cholangioscope-assisted ERAT were (with written parental consent) performed ( Video 1Video 1 ). Colonoscopy revealed the appendiceal orifice was normal ( Fig. 2Fig. 2 **a** ). A cholangioscope was used to intubate the appendiceal cavity, and purulent fluid rapidly flowed out ( Fig. 2Fig. 2 **b** ). With a clear direct view through the cholangioscope, an appendicolith measuring approximately 2.5 cm in size was discovered in the appendiceal cavity ( Fig. 3Fig. 3 **a** ); the intraluminal mucosa was congested and edematous with white pus adhesions ( Fig. 3Fig. 3 **b** ). The appendix was adequately irrigated with normal saline, and the appendicolith was successfully expelled from the cavity ( Fig. 4Fig. 4 ). The patient reported his abdominal pain to be relieved after the cholangioscope-assisted ERAT.

**Fig. 1:**
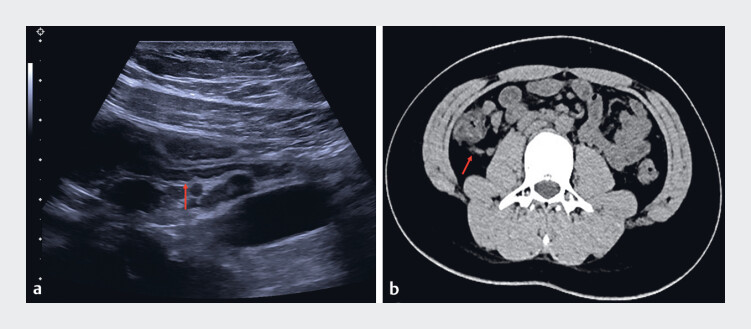
**Fig. 1** Imaging studies showed the appendix was normal: **a** ultrasound; **b** low-dose CT.

**Fig. 2:**
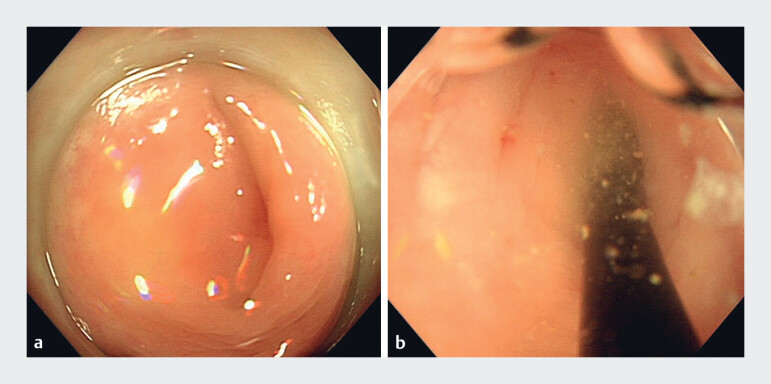
**Fig. 2a** Colonoscopy revealed a normal appendiceal orifice. **b** On cholangioscope intubation, purulent fluid flowed out of the appendiceal cavity.

**Fig. 3:**
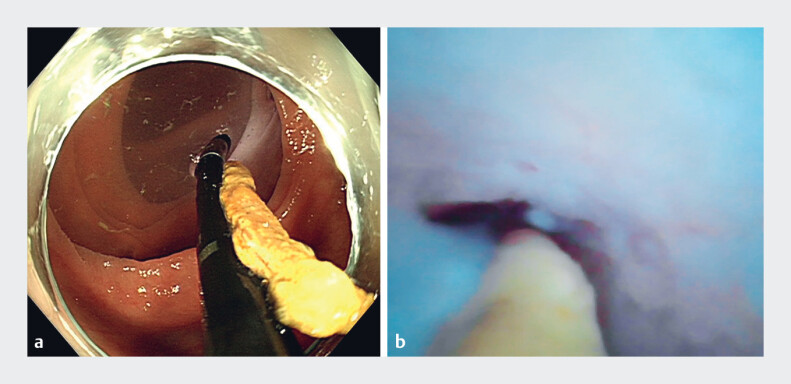
**Fig. 3** Cholangioscopic views. **a** A giant appendicolith, approximately 2.5 cm in size (the thin white scale bars on the cholangioscope indicate 1 cm); **b** congested and edematous intraluminal mucosa.

**Fig. 4:**
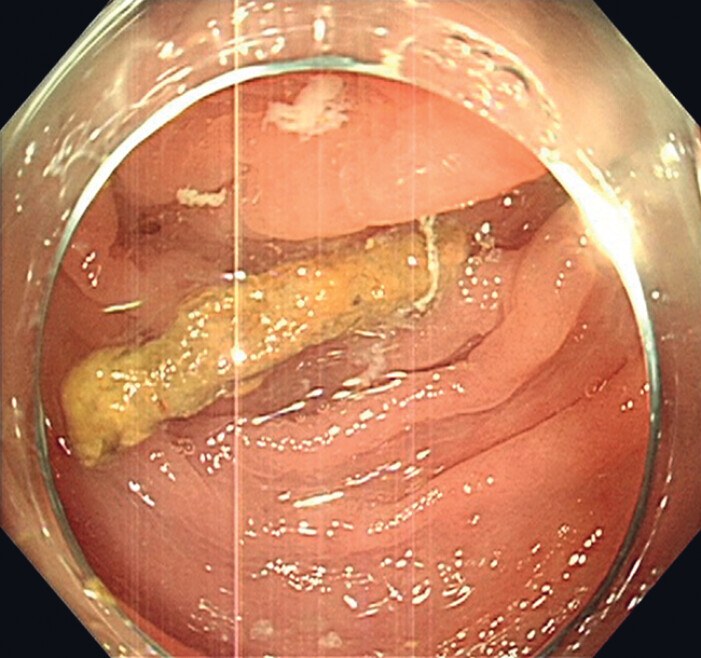
**Fig. 4** The appendicolith was successfully expelled from the cavity.

Cholangioscope-assisted endoscopic retrograde appendicitis therapy used to diagnose and treat occult appendicitis with a giant fecalith.Video 1Video 1

Endoscopy_UCTN_Code_TTT_1AQ_2AH

## Notes

Endoscopy E-Videos https://eref.thieme.de/e-videos
*E-Videos* is an open access online section of the journal *Endoscopy* , reporting on interesting cases and new techniques in gastroenterological endoscopy. All papers include a high-quality video and are published with a Creative Commons CC-BY license. Endoscopy E-Videos qualify for HINARI discounts and waivers and eligibility is automatically checked during the submission process. We grant 100% waivers to articles whose corresponding authors are based in Group A countries and 50% waivers to those who are based in Group B countries as classified by Research4Life (see: https://www.research4life.org/access/eligibility/ ). This section has its own submission website at https://mc.manuscriptcentral.com/e-videos .

## References

[1] Ranieri DM Enzerra MD Pickhardt PJ Prevalence of appendicoliths detected at CT in adults with suspected appendicitis AJR Am J Roentgenol 2021 216 677 682 10.2214/AJR.20.23149 33474985

[2] Ishiyama M Yanase F Taketa T Significance of size and location of appendicoliths as exacerbating factor of acute appendicitis Emerg Radiol 2013 20 125 130 10.1007/s10140-012-1093-5 23179506

[3] Kong LJ Liu D Zhang JY Digital single-operator cholangioscope for endoscopic retrograde appendicitis therapy Endoscopy 2022 54 396 400 10.1055/a-1490-0434 33893629

